# Crystallization of Polytetrafluoroethylene in a Wide Range of Cooling Rates: Nucleation and Diffusion in the Presence of Nanosilica Clusters

**DOI:** 10.3390/molecules24091797

**Published:** 2019-05-09

**Authors:** Nicolas Bosq, Nathanaël Guigo, Jacques Persello, Nicolas Sbirrazzuoli

**Affiliations:** 1 Université Côte d’Azur, Institut de Chimie de Nice, UMR CNRS 7272, 06100 Nice, France; nicolas.bosq@univ-cotedazur.fr; 2 Université Côte d’Azur, Institut de Physique de Nice, UMR CNRS 7010, 06100 Nice, France; jacques.persello@univ-cotedazur.fr

**Keywords:** polytetrafluoroethylene, silica, nanocomposite, crystallization kinetics, isoconversional analysis

## Abstract

Polytetrafluoroethylene (PTFE) is a polymer that displays exceptional properties. This synthetic fluoropolymer is also known to crystallize very fast upon cooling. The present work highlights for the first time the influence of nanosilica clusters on PTFE crystallization at fast cooling rates (up to 5000 K·s^−1^). The silica was synthesized from aqueous silicate solution and the surface modification was performed using TriEthoxyFluoroSilane (TEFS). In order to understand the crystallization behavior of PTFE/silica nanocomposite at a fast cooling rate, the measurements were carried out by Fast Scanning Calorimetry (FSC). The data were consequently combined with the measurements performed by conventional Differential Scanning Calorimetry (DSC). Interestingly, the results displayed variation of the crystallization behavior for the nanocomposite at fast cooling rates compared to slow cooling rates. The differences in crystal morphologies were then observed by Scanning Electron Microscopy (SEM) after slow and fast cooling rates. Finally, the effective activation energies (*E*_α_) obtained from the crystallization under various cooling rates were combined in order to obtain one set of Hoffman-Lauritzen parameters. This procedure allowed us to show that the crystallization of PTFE in the presence of silica is promoted or hampered according to the cooling rates employed.

## 1. Introduction

Polytetrafluoroethylene (PTFE) is a well-known synthetic thermoplastic fluoropolymer. This polymer was originally discovered in 1938 by Roy Plunkett of E.I. du Pont de Nemours and Company. The chemical structure of PTFE consists of a carbon skeleton surrounded by a protective layer of fluorine atoms. The presence of fluorine atoms confers to the PTFE a good resistance to chemical or thermal actions/damage additionally to outstanding surface and interface properties. Its good chemical inertness, thermal stability, hydrophobicity, and biocompatibility properties allow the PTFE to be employed for the elaboration of smart materials exposed to extreme conditions [1]. Consequently, this polymer is widely used for aeronautics [2] and aerospace applications [3,4]. Due to its interesting properties, domestic and industrial applications for this polymer are also numerous [5]. As an example, PTFE appears to be well-suited for cable insulations and microwave applications by displaying excellent insulating and dielectric properties. Additionally, PTFE can be used at high temperatures since this polymer displays a melting point at ~327 °C while its low coefficient of friction and high rate of wear allow this polymer to be suitable for bearing and seals applications [6].

Several of the macroscopic properties of PTFE-based materials, and particularly its thermomechanical behavior, are determined by its microstructure. Since the microstructure of a semicrystalline polymer is tightly linked to the degree of crystallinity and the crystal morphology [7,8] the investigation of PTFE crystallization kinetics has to be carefully investigated. The neat PTFE displays a high degree of structural regularity that induces a very fast crystallization compared to other semi-crystalline polymers [9]. As the PTFE crystallizes very fast, the conventional experimental solutions to analyze the crystallization process of PTFE-based materials are considerably limited. Differential Scanning Calorimetry (DSC) allows the employment of cooling rates up to 1 K.s^−1^ and can be routinely used to record experimental data [10]. However, faster cooling rates can be employed via Fast Scanning Calorimetry (FSC) and were employed to study the crystallization of polymers such as polyamide, [11] poly-caprolactone [12,13], and iPP [14,15], providing innovative information with suppressing partially or entirely the ordering process. Using the calorimeter chip technology, this technique combines the minuscule amount of sample with the small thermal resistance of the chip in order to reach fast heating and cooling rates (up to 5000 K·s^−1^). FSC was also employed to observe the crystallization process of PTFE over a wide temperature range [9]. The data collected consequently helped to obtain new knowledge on this polymer crystallization behavior, in particular on the impossibility to bypass PTFE crystallization under fast cooling (up to 800,000 K·s^−1^), as well as new transitions in the crystallization kinetic regime.

Great attention has been paid during the past few years to the variations of properties induced by matrix/filler interaction in hybrid materials. Several studies have been performed on PTFE hybrid materials, and many authors investigated the effect of an inorganic filler inserted in this fluoropolymer, such as ceramic and micro-fiberglass particles [16,17,18]. It has been shown that the glass fibers induced a poor nucleation activity [10], and it was highlighted that the presence of CaCO_3_ allows the one-dimensional growth of crystals generated from inorganic fillers [19]. However, the secondary crystallization was not observed with the insertion of fillers, but according to a previous study, the presence of solid glass microspheres also promoted the heterogeneous nucleation process. [20] In addition, several research activities also focus on silica fillers. These investigations show that the insertion of silica into PTFE appears to be of real interest since it allows to improve the mechanical properties but also the dielectric constant of this polymer [21]. Based on this result, Martins et al. [22,23] evaluated the mechanical properties of PTFE/silica composite and observed an increase of elastic modulus and stiffness in the presence of the filler. Madani et al. [24] highlighted the variations of PTFE/silica microstructure according to the silica concentration. Additionally, Beckford et al. [25] studied the effect of silica on PTFE properties such as friction and wear resistance. Another study investigated the effect of the insertion of two different sized silica (5 µm and 20 nm) on the PTFE thermomechanical properties [26]. As for composites and nanocomposites in general, good compatibilities and good interface are required to obtain significant variations of properties attributed to the filler. Consequently, Chen et al. [21]. studied the effect of phenyltrimethoxysilane coupling agents in order to improve the properties of PTFE/silica composites. In opposition, untreated silica lead to materials with poor mechanical properties, high porosity, and high water uptake [27,28]. The silica surface modification performed with fluoroalkysilane allowed thus to obtain PTFE composites with higher levels of performance, such as improvement of flexural endurance and ductility [29]. Indeed, such surface fluorination helped the decrease of the silica hydrophilicity and helped to reduce the void. It has to be noticed here that the effect of non-modified silica filler on PTFE crystallization was already studied for conventional cooling rates [30] and that the work mentioned before focuses on the composite final properties. However, to our knowledge, no studies have investigated the effect of fluorinated nanosilica clusters on PTFE crystallization at fast cooling rates. Since this field remains poorly described, it is consequently essential to investigate both at slow and fast cooling rates the effect induced by a silica presenting a specific morphology and displaying appropriate chemical functions on its surface.

The originality of the present study lies in the fact that the PTFE crystallization is investigated simultaneously under a wide range of cooling rates and in the presence of a silica presenting an atypical morphology (i.e., nanosilica clusters). First, fluorination of the filler was performed to improve its compatibility with the matrix. This modification was confirmed via Fourier Transform Infrared Spectroscopy (FTIR) and solid-state Nuclear Magnetic Resonance spectroscopy (NMR). The filler dispersion into PTFE was evaluated by Transmission Electron Microscopy (TEM). Then, the crystallization behavior of PTFE nanocomposite was investigated by both conventional DSC and FSC. Scanning Electron Microscopy (SEM) was also employed to observe the morphology of the crystals formed under various cooling rates. Additionally, the thermoanalytical data collected in a wide range of temperature (DSC and FSC data) were computed by an advanced isoconversional method in order to obtain the effective activation energy of crystallization (*E*_α_). Finally, Hoffman-Weeks [31] theory was applied to calorimetry data in order to estimate the equilibrium melting temperature (*T*^0^_m_), and Hoffman-Lauritzen [32] theory was applied to *E*_α_ in order to investigate the nucleation and the diffusion of crystal formed.

## 2. Results and Discussion

### 2.1. Characterization of Silica Surface Modification

**Figure 1:**
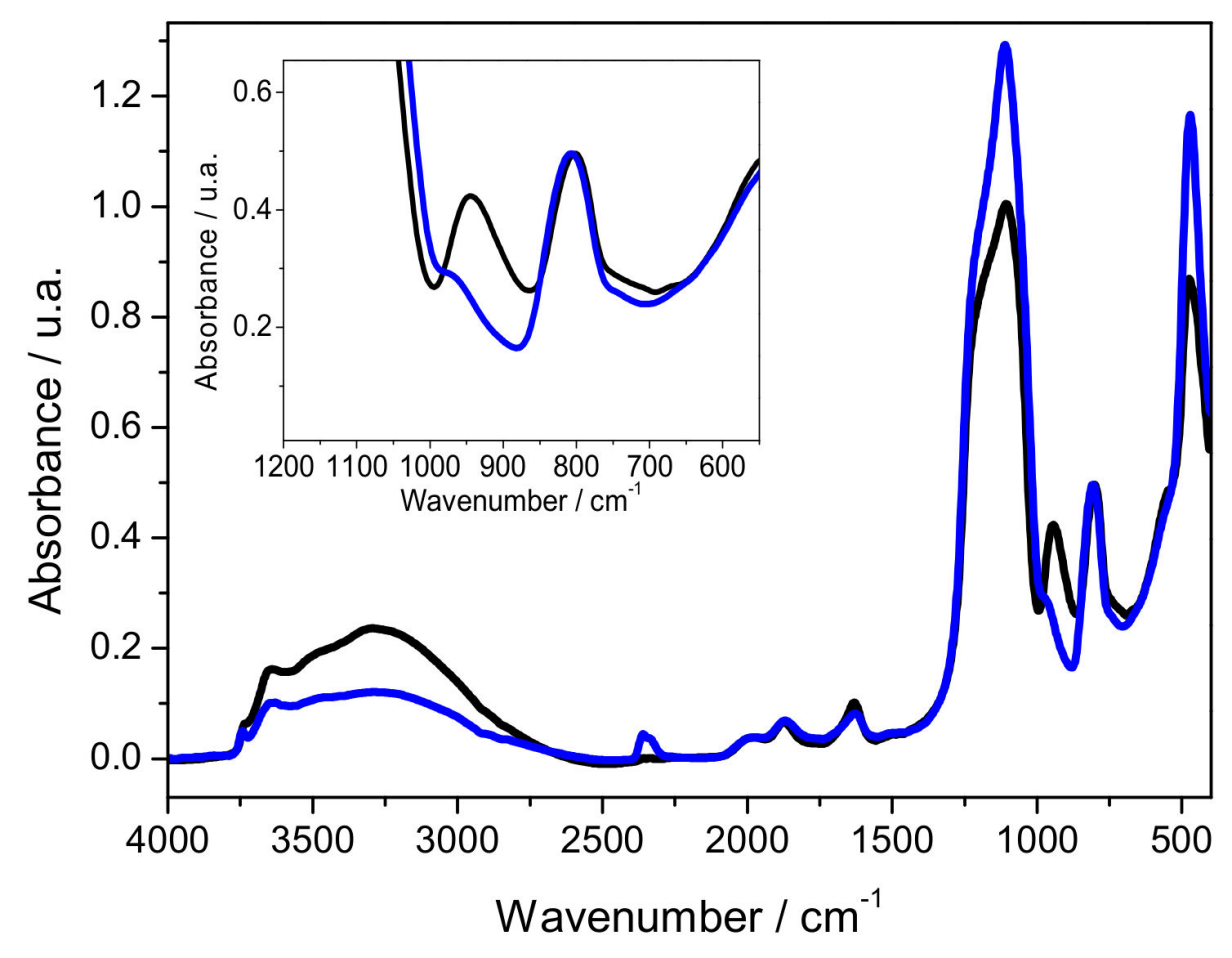
FTIR spectra of nanosilica clusters. Black: SiO_2_(c). Blue: SiO_2_(c)F. Inset: Magnification of FTIR spectra in the temperature range of 1200–550 cm^−1^.

FTIR spectroscopy was performed to verify the surface modification of silica. The FTIR spectra of SiO_2_(c) and SiO_2_(c)F are presented in Figure 1.

The minimal value was fixed at 0 in a non-absorbing area (2250 cm^−1^) and data were normalized at 1 from the value at 1110 cm^−1^ corresponding to the band of silica Si-O-Si antisymmetric stretching vibration [33]. A substantial decrease of the OH-band between 3600 and 3000 cm^−1^ is observed on the modified silica spectrum. This result shows the decrease of the free O-H groups amount on the silica surface after the functionalization [34]. Finally, the variation of the band intensity at ~950 cm^−1^ as presented in the inset of Figure 1 and corresponding to the stretching vibration of Si-F groups confirms the presence of fluorine atoms on nanosilica clusters surface [35].

**Figure 2:**
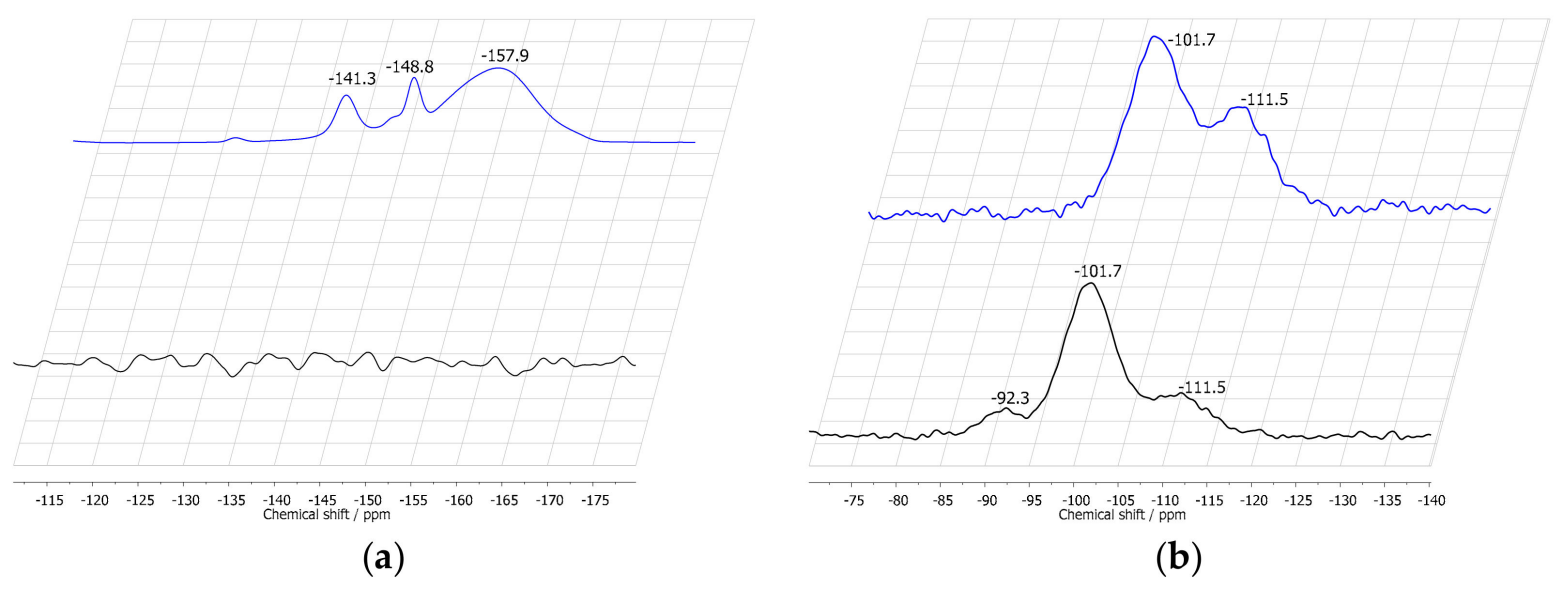
CP/MAS ^19^F (**a**) and CP/MAS ^29^Si (**b**) solid-state NMR spectra. Black line: SiO_2_(c). Blue line: SiO_2_(c)F. All spectra were collected at a spin rate of 10 kHz. The chemical shift value at the top of the peak is indicated by the curve.

Significant structural variations associated with the modification of the silica surface are observed via solid-state NMR analysis. The CP/MAS ^19^F and CP/MAS ^29^Si spectra of SiO_2_(c) and SiO_2_(c)F are depicted on Figure 2.

The CP/MAS ^19^F spectrum of SiO_2_(c)F displays typical resonances of fluorine functions. According to the work of Lataste et al. [36], the resonance at ~−158 ppm is attributed to isolated O_3/2_SiF entities, the resonance merged in the broad band at ~−153 ppm corresponds to O_4/2_SiF entities and the resonance at ~−149 ppm is characteristic of O_3/2_SiF entities located close to other groups of the same type. Additionally, the resonance at ~−141 ppm is attributed to O_3/2_SiF_2_ entities. This confirms consequently the presence of fluorine functions onto SiO_2_(c)F surface. The resonances from CP/MAS ^29^Si spectrum of SiO_2_(c) located at ~−112, −102 and −92 ppm are associated with O_4/2_Si, O_3/2_SiOH and O_2/2_Si(OH)_2_ entities, respectively [33]. The resonances corresponding to O_4/2_Si entities and O_3/2_SiOH entities are also displayed on SiO_2_(c)F spectrum, but the resonance corresponding to O_2/2_Si(OH)_2_ entities is not apparent. Additionally, the resonance of O_4/2_Si entities is more intense for SiO_2_(c)F, meaning that the SiOH/Si-O-Si ratio of the modified silica is lower compared to the neat silica. These results confirm the substitution of hydroxyl groups by fluorine atoms during the surface modification.

The results mentioned above from complimentary techniques are correlated and lead to the conclusion that the silica was successfully modified with fluorine atoms covalently bonded on its surface.

### 2.2. Morphology of PTFE/SiO_2_(c)F Nanocomposite

**Figure 3:**
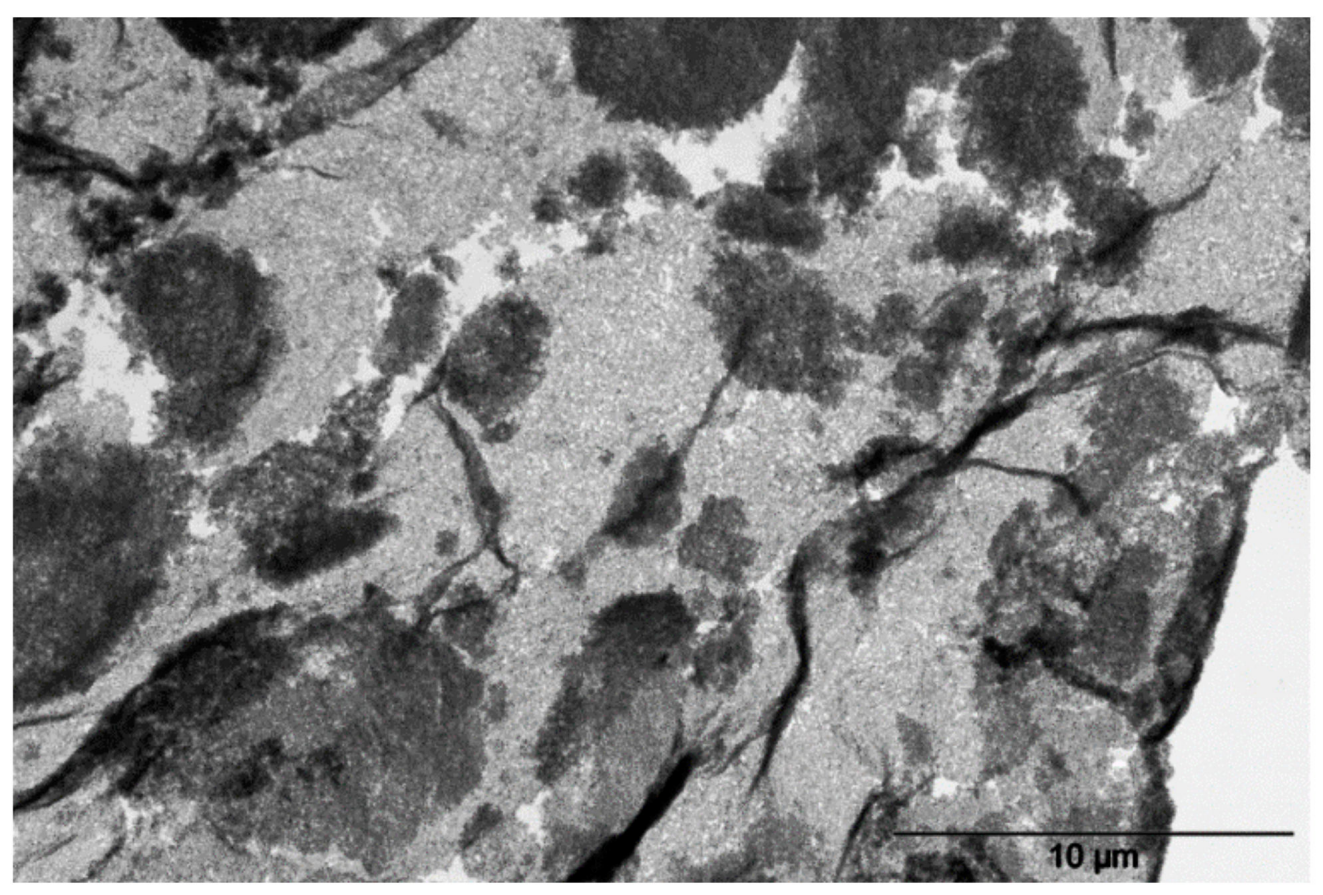
TEM picture of the PTFE/SiO_2_(c)F nanocomposite at a 10 µm scale.

The TEM picture of PTFE/SiO_2_(c)F nanocomposite is displayed in Figure 3.

It is worth noting that the black spots appearing on the picture are due to cohesion rupture occurring during ultramicrotome cutting [37]. They are consequently not representative of the nanocomposite internal structure. As observed at low magnitude, there is no evidence of aggregation, and the modified nanosilica clusters are homogeneously dispersed into the PTFE matrix. This homogeneous dispersion is due to the surface modification of silica that promotes the compatibility of the filler with the polymer matrix. Indeed, the modified silica displays hydrophobic chemical groups on its surface and is dispersed in a hydrophobic polymer matrix. The homogenous dispersion of the modified silica into the PTFE consequently results in homogeneous properties for the material.

### 2.3. Crystallization Kinetics of Neat PTFE and PTFE/SiO_2_(c)F

#### 2.3.1. Melt Crystallization

**Figure 4:**
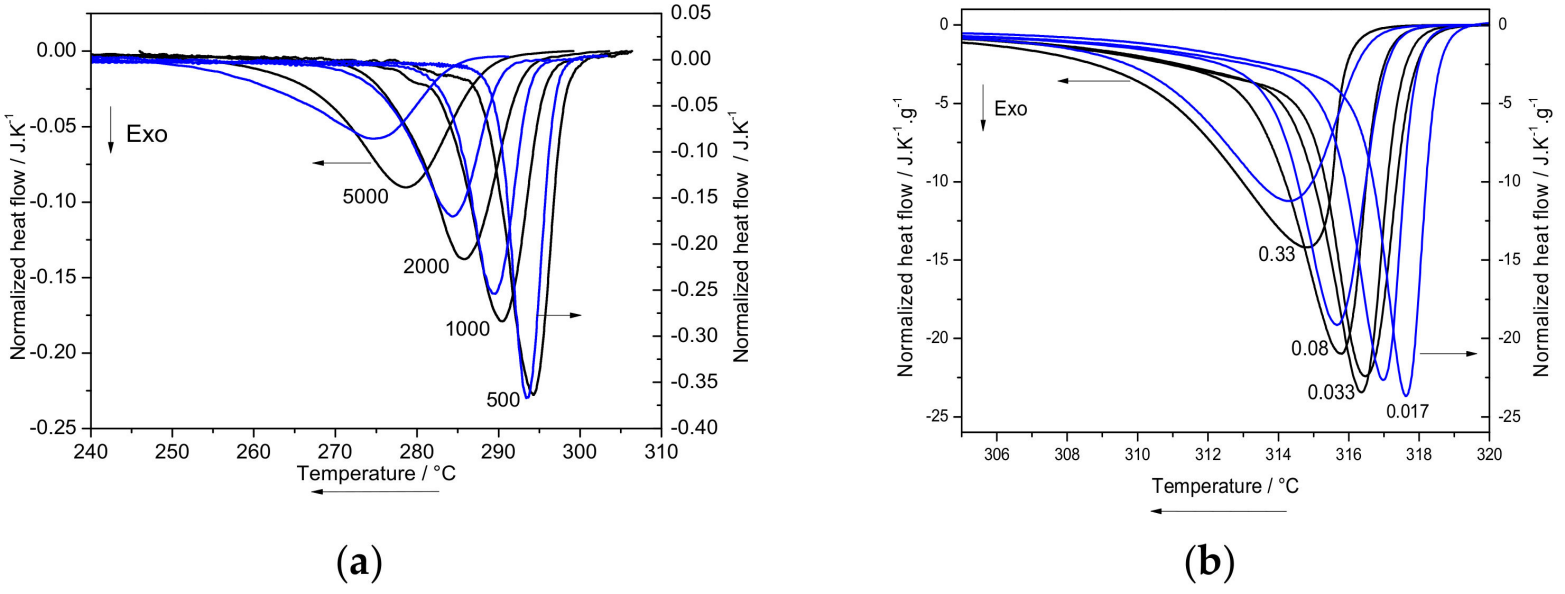
FSC (**a**) and DSC (**b**) heat flow normalized to cooling rate for non-isothermal crystallization from the melt at different cooling rates, considering samples with a mass of ~27 ng and ~4 mg respectively. Black: PTFE, blue: PTFE/SiO_2_(c)F. The cooling rate of each experiment (in K·s^−1^) is indicated by each curve.

Figure 4 shows the normalized heat flow curves measured by non-isothermal DSC and FSC scans during crystallization from the melt.

**Figure 5:**
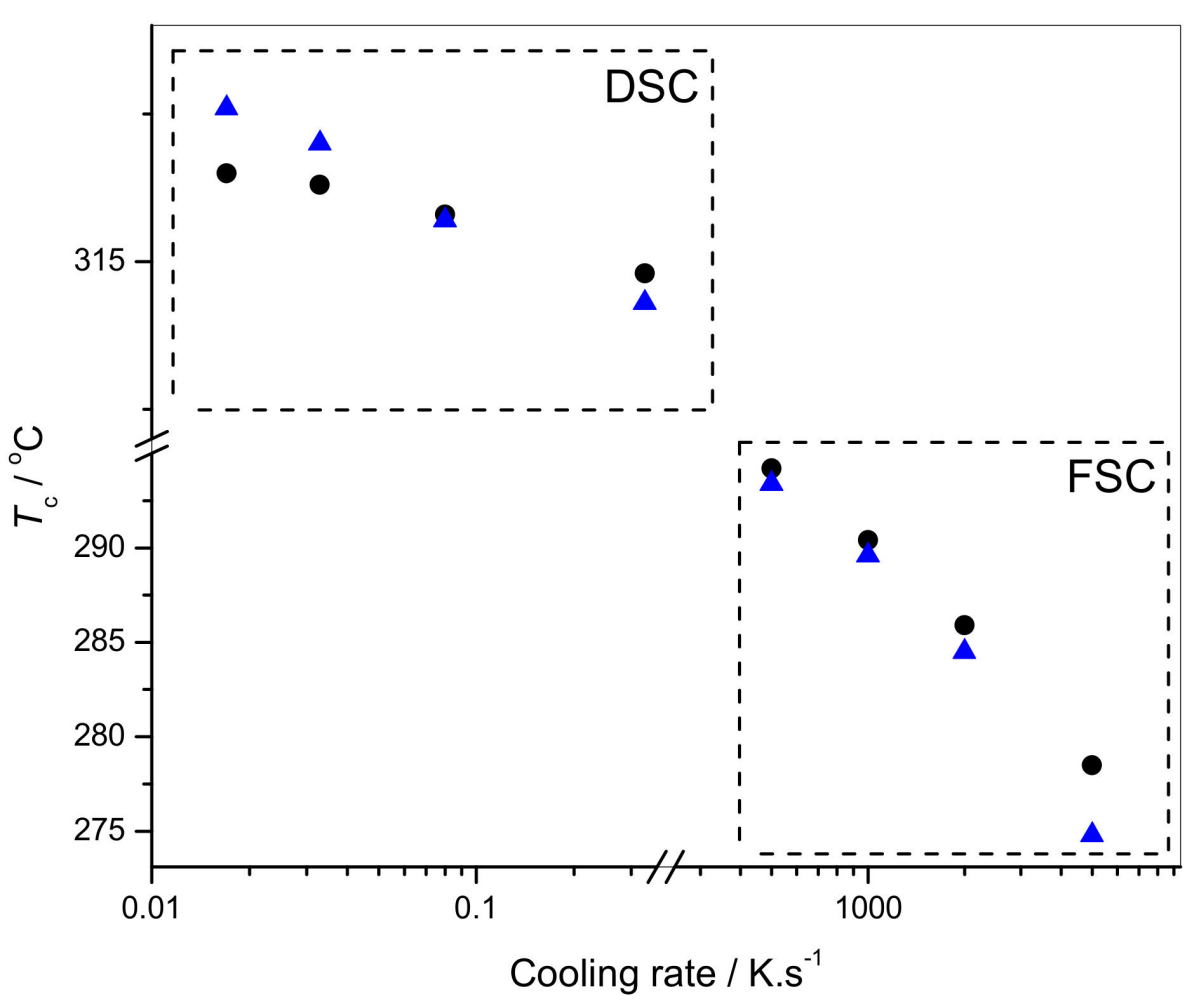
Crystallization temperatures obtained by DSC and FSC for non-isothermal crystallization from the melt at different cooling rates. Black dots: Neat polytetrafluoroethylene (PTFE); blue triangles: PTFE/SiO_2_(c)F.

The thermoanalytical curves represent the data obtained at slow and fast cooling rates during DSC and FSC measurements. According to the results, the crystallization peak is normally shifted to a lower temperature with increasing cooling rate. Besides, it appears for both samples that the exothermal crystallization peak is not symmetrical for slow cooling rates. This asymmetry can be explained by a transition from a primary to a secondary crystallization process [20,38,39]. Yet, it must be stressed that the peaks remain symmetric at fast cooling rates, indicating that the secondary crystallization process is limited in this case. This indicates that the crystals formed under fast cooling rates cannot reach a sufficient development of their structure and are not able to start the secondary crystallization process. Indeed, we have previously shown that PTFE crystals formed under fast cooling present one single melting peak contrary to crystals formed under sufficiently slow cooling which presented a well-marked shoulder. It suggested that fast cooling did not permit secondary crystallization to occur. These PTFE crystals formed under fast cooling presented an axialite morphology which does not favor the secondary crystallization as the lamellae appear to be thinner and do not have sufficient time to form perfectly, [40] contrary to spherulite or ribbon morphology that present thicker lamellae. The variations of *T*_c_ displayed in Figure 4 between the two samples present similar amplitudes as the variation amplitudes of *T*_c_ observed in the study of Smith et al. [41] on Poly(trimethylene terephthalate) that were employed for kinetics analysis. Figure 5 displays the values of *T*_c_ measured by non-isothermal DSC and FSC measurements during crystallization from the melt for the two samples.

The DSC curves obtained in Figure 4 and the values of *T*_c_ displayed on Figure 5 clearly show that the crystallization of PTFE/SiO_2_(c)F from the melt occurs at higher temperature compared to the neat PTFE in the temperature range where the crystallization is limited by the nucleation (i.e., close to the equilibrium melting temperature). This result observed for the slowest cooling rates can be justified by the promotion of nucleation induced by the presence of nanosilica clusters. Several studies employing micrometric silica mention that this filler does not modify the crystallization of PTFE [29] and also can hinder it according to the silica content inserted [30]. However, the authors did not investigate the effect of their filler at very low cooling rates with regular DSC as presented here. Besides, Figure 4 and Figure 5 show that the crystallization of the nanocomposite performed under fast cooling rates occurs at lower temperatures compared to the neat PTFE. The crystallization temperatures corresponding to the crystallization performed under fast cooling rates tend to be located further from *T*_m_ where the nucleation becomes less determining. Consequently, the nucleating effect induced by the silica is less pronounced in this temperature range and the presence of nanosilica clusters hinders the diffusion of polymer chains during the crystal growth (i.e., at temperatures much lower than *T*_m_). These results are corroborated with the data of Figure S2 (see Supplementary Materials). Indeed, the temperatures at which the crystallization starts to become significant (*T*_onset_) are higher in the presence of silica for low cooling rates. However, *T*_onset_ also appears to be lower in the presence of silica for high cooling rates. The half-crystallization time (*t*_1/2_) was evaluated from the data of Figure S2 and shows that the values of *t*_1/2_ are lower for the nanocomposite at low cooling rates, corresponding to an increase of the crystallization rate defined by dα/dt in the presence of silica.

#### 2.3.2. Crystal Morphology of PTFE/SiO_2_(c)F

**Figure 6:**
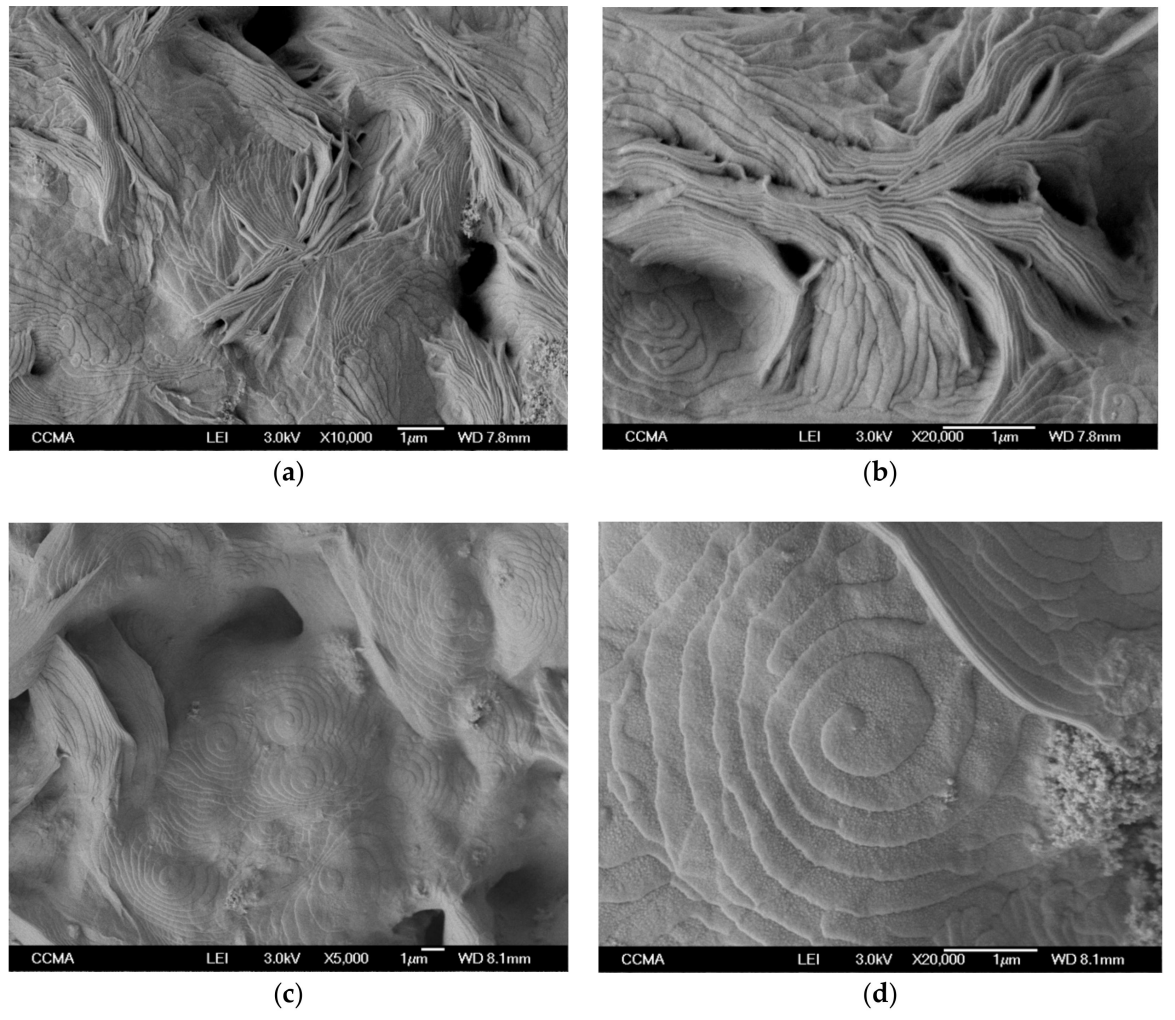
SEM micrograph of PTFE crystals formed in the presence of SiO_2_(c)F at (**a**,**b**) fast cooling rate (100 K·s^−1^) and (**c**,**d**) slow cooling rate (0.1 K·s^−1^).

In order to investigate the influence of the nanosilica clusters on the PTFE crystal morphology, SEM observations were performed on PTFE/SiO_2_(c)F. The crystalline structure of PTFE/SiO_2_(c)F obtained after fast and slow cooling from the melt is presented in Figure 6.

The PTFE/SiO_2_(c)F crystalline morphology formed from the melt after a cooling rate of 100 K·s^−1^ is displayed in Figure 6a,b. It can be observed from the micrographs that several crystals present a rod-like or needle-like shape. However, compared to the results obtained for the neat PTFE [9], two-dimensional crystallites have also developed and display a disc-like shape. This shows that the nanosilica clusters promote the nucleation and the diffusion of crystal chains with the formation of two-dimensional structures even under relatively fast cooling rates. The PTFE/SiO_2_(c)F crystalline morphology formed from the melt after a cooling rate of 0.1 K·s^−1^ is presented in Figure 6c,d. The micrographs clearly show that the crystals exhibit ribbon-like structures arranged into concentric discs or spirals. These structures formed at the slow cooling rate are obviously different from the rod-like structure observed after the fast cooling rate. Compared to the crystals of neat PTFE [9], the crystals in the nanocomposite display disc structures with a smaller diameter but are more numerous. This attests that the number of nucleation centers is enhanced by the presence of nanosilica clusters and confirms the promotion of PTFE nucleation.

#### 2.3.3. *E*_α_ Dependences and Evaluation of Hoffman-Lauritzen Parameters

**Figure 7:**
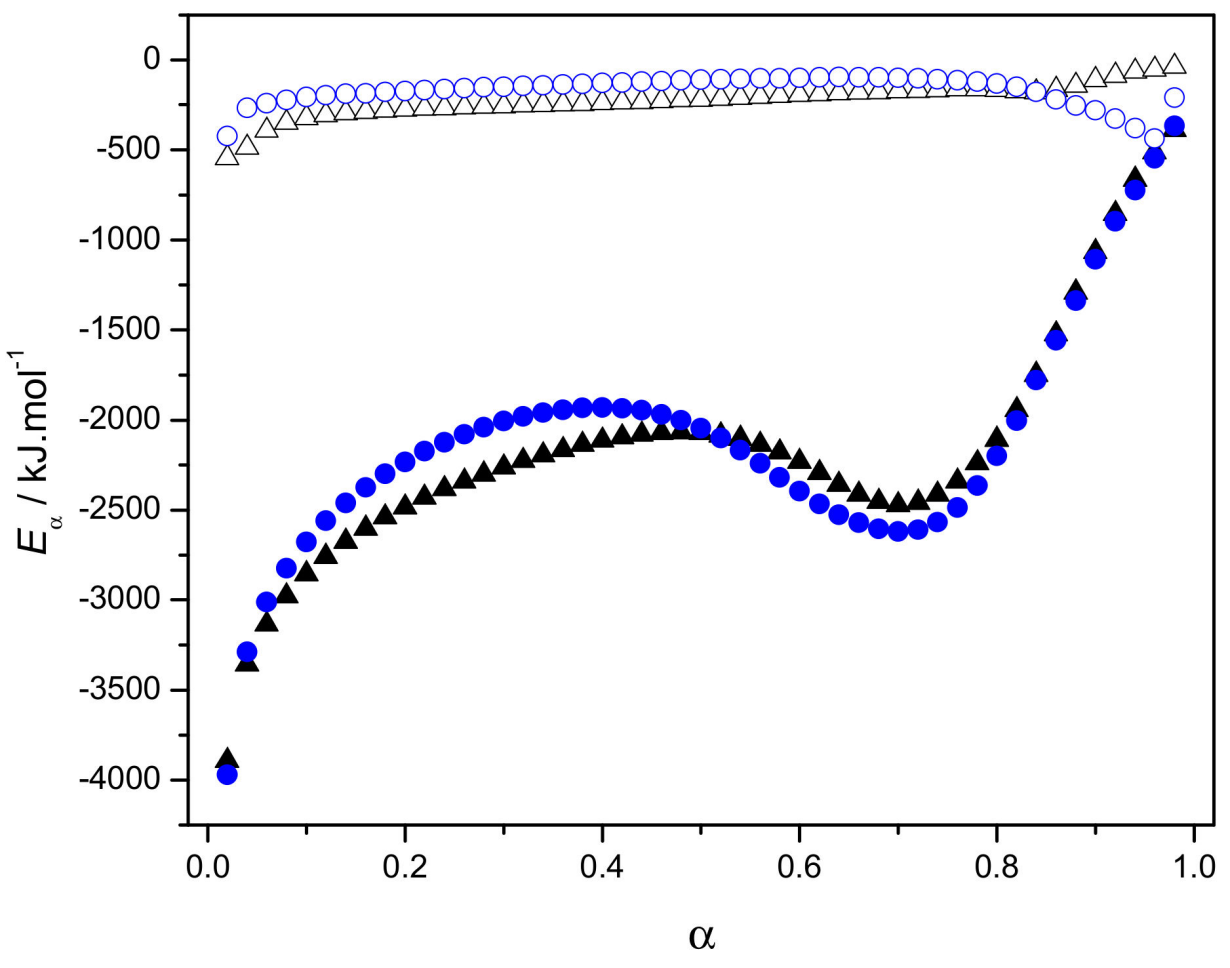
*E*_α_ dependence vs. relative degree of crystallinity computed from FSC and DSC data. Black triangles: PTFE; blue circles: PTFE/SiO_2_(c)F; open: Crystallization performed at the cooling rates 500, 1000, 2000 and 5000 K·s^−1^; solid: Crystallization performed at the cooling rates 0.0167, 0.0333, 0.0833, and 0.3333 K·s^−1.^

The advanced isoconversional kinetic analysis was applied to the non-isothermal crystallization of neat PTFE and PTFE/SiO_2_(c)F measured with both regular DSC and FSC. The calculation was performed using the data presented in Figure 4. This procedure allows plotting of the dependency of the effective activation energy of crystallization with the relative degree of crystallinity (*α*). The result of the dependencies is presented in Figure 7, where each value of *E*_α_ corresponds to a certain interval of temperature [42].

The existence of this dependence reflects the complexity of the crystallization process, which is a multiple-step process that depends on both the relative extent of crystallization and temperature. In this case, the definition of an energy barrier cannot be attributed to the effective activation energy of crystallization, and *E*_α_ has to be rather attributed to the temperature dependence of temperature coefficient of the crystallization rate. The *E*_α_ dependence on α in Figure 7 displays a similar crystallization process for the two materials. As for the neat PTFE, the *E*_α_ curve of the nanocomposite shows negative values that globally increase on whole range of α. This indicates that the overall crystallization rate is controlled by nucleation. A similar behavior was observed for poly(ethylene terephthalate) (PET) [43], poly(butylene succinate) [44] and poly(vinylidene fluoride) [45] crystallization. This variation corresponds to an anti-arrhenian behavior of PTFE and PTFE/SiO_2_(c)F crystallization from the melt. Nevertheless, a decrease is observed for α around 0.50. This decrease is not in agreement with what is observed by applying an advanced isoconversional method to the Hoffman-Lauritzen equation [46]. Thus, interpretation of the *E*_α_-dependency for the complex crystallization of PTFE is not straightforward, but it can be concluded that additional phenomena not taken into account in the secondary crystallization theory of Hoffman and Lauritzen occurs in this region. Although fast cooling rates are employed, no positive values of *E*_α_ corresponding to an Arrhenius behavior are observed in the experimental temperature range investigated. This result shows that the crystallization on the cooling of neat PTFE and PTFE/SiO_2_(c)F cannot be suppressed. Thus, the crystallization on heating from the glass and its corresponding *E*_α_–dependency cannot be observed, as it was the case for PET [47,48].

A cut break at *α* ~0.70 corresponding to an *E*_α_ value of −2610 kJ·mol^−1^ is clearly observed on the graph. This break was previously associated to the transition from crystallization regime II to regime III [9]. This result is consistent with the work of Vyazovkin et al. [43] who observed similar behavior for PET crystallization. During regime II, the substrate completion rate and the surface nucleation rate reach approximately the same order, whereas regime III is characterized by a surface nucleation rate that is very fast. In regime III, the rate of crystal growth is consequently governed by the substrate completion rate. It is important to highlight here that the *E*_α_ curve of PTFE/SiO_2_(c)F presents a cut break at *α* ~0.70 similar to the cut break of neat PTFE. This result shows that the presence of nanosilica clusters also allows it to preserve the transition between the crystallization regimes II and III.

**Figure 8:**
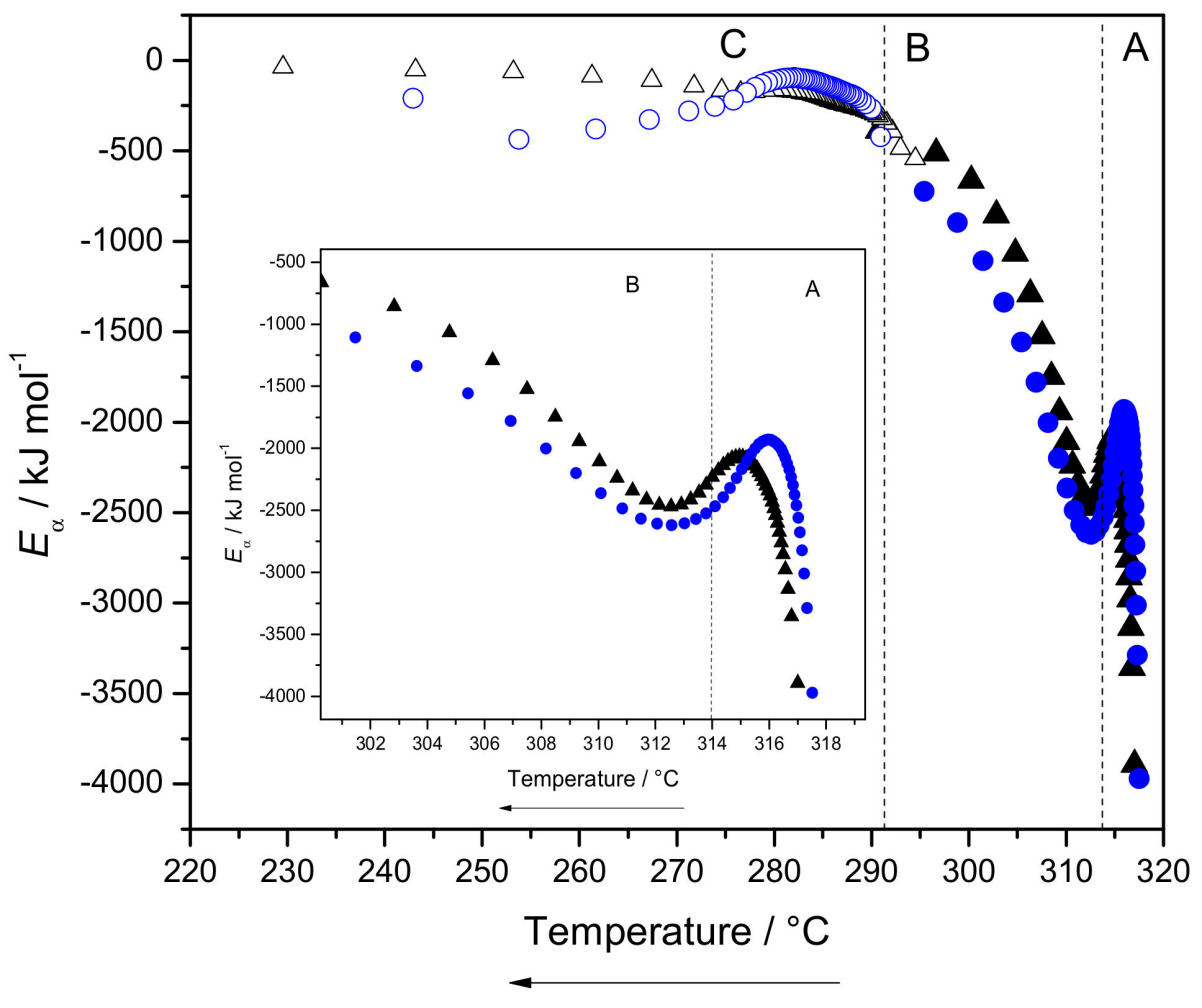
*E*_α_ dependence vs. temperature computed from FSC and DSC data. Black triangles: PTFE; blue circles: PTFE/SiO_2_(c)F; open: Crystallization performed at the FSC cooling rates 500, 1000, 2000 and 5000 K·s^−1^; solid: Crystallization performed at the DSC cooling rates 0.0167, 0.0333, 0.0833, and 0.3333 K·s^−1^. Inset: Magnification of *E*_α_ vs. *T* curve in the temperature range of 300–320 °C.

Figure 8 shows the *E*_α_ vs. *T* dependencies obtained from the melt crystallization of neat PTFE and PTFE/SiO_2_(c)F.

The *E*_α_ values computed from DSC and FSC measurements display a good continuity for the two samples. Indeed, it clearly appears that the last *E*_α_ value of DSC is correlated with the first *E*_α_ value of FSC, and the evaluation effective activation energy can be so performed on a large temperature range (~90 °C). Several variations of *E*_α_ curve are observed in Figure 8 and are correlated to the variations observed on *E*_α_ vs. α dependency. Each variation corresponds to a change in the overall crystallization mechanism of the PTFE matrix and allows for separation of the *E*_α_ dependency in three different regions that are similar for the neat polymer and the nanocomposite. This result shows that the mechanisms involved in the overall crystallization of PTFE matrix are similar in the presence of nanosilica clusters. The first region (A) is located between 320 and 312 °C. In this region, the *E*_α_ values of neat PTFE and PTFE/SiO_2_(c)F tend first to increase for 320 < T < 315–316 °C, close to the equilibrium melting temperature *T*^0^_m_ ~324 °C, then to decrease for the temperatures between 315–316 °C and 312–313 °C. This can be explained by the formation of a stable nuclei that is rate-determining. A break is obviously present at ~312 °C for both samples corresponding to a transition between two crystallization kinetics regimes and shows a change in the crystallization mechanism. The amplitude of this break for the nanocomposite is similar to the amplitude observed for the neat polymer and corresponds to the transition between crystallization mechanisms II and III, as observed on the *E*_α_ vs. α dependency [9]. This result confirms that the presence of nanosilica clusters induces a nucleating effect in this temperature region and simultaneously allows it to preserve the kinetic regime II and III. The second region (B) is located between 312 and 290 °C. The temperatures in this region are further from *T*^0^_m_ so the crystallization is governed by crystal growth as the nucleation occurs faster. The third region (C) is located below 290 °C, these temperatures are far from *T*^0^_m_, the nucleation rate is then very fast and is less determining compared to the rate of surface nucleus spread process. According to Figure 8, the *E*_α_ values of PTFE/SiO_2_(c)F globally increase with decreasing temperature on the whole temperature range. However, the local variations of *E*_α_ observed for PTFE/SiO_2_(c)F allow it to corroborate the results with the morphology of crystals depicted in Figure 6. The region (C) can be associated to the crystals formed under very fast cooling rate that display mostly one-dimensional rod-like structures. In this region, the *E*_α_ values of PTFE/SiO_2_(c)F observed at ~250 °C tend to be lower than the *E*_α_ values of neat PTFE. Finally, regions A and B are associated with the crystals formed at the slow cooling rate, where the disk-like morphology is consequently promoted.

**Figure 9:**
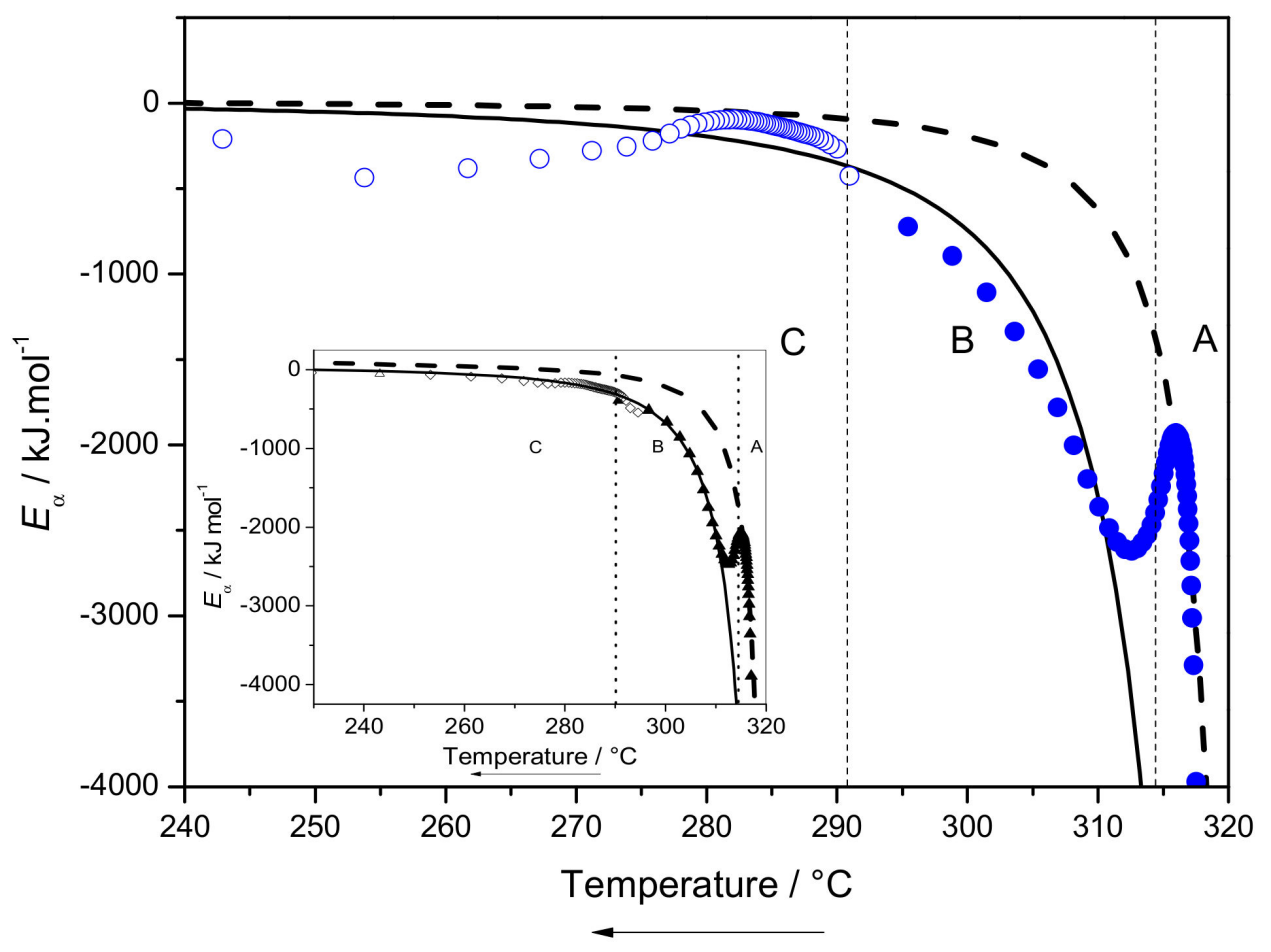
Fit of *E*_α_ dependence vs. temperature for PTFE/SiO_2_(c)F and PTFE (inset) samples. Black triangles: PTFE; blue circles: PTFE/SiO_2_(c)F; open: Crystallization performed at the FSC cooling rates 500, 1000, 2000, and 5000 K·s^−1^; solid: Crystallization performed at the DSC cooling rates 0.0167, 0.0333, 0.0833, and 0.3333 K·s^−1^. Non-linear fits performed via Equation (8) for region B−C (line) and region A−C (dash) are shown.

*E*_α_ vs. *T* dependencies of PTFE and PTFE/SiO_2_(c)F were fitted to Equation (8), and the non-linear fits are depicted in Figure 9 for both samples.

In order to avoid the divergence of the diffusion parameter, U* was fixed to 6270 J·mol^−1^ according to previous studies [10,47]. The values *T*_m_ = 597 K, and *T*_∞_ = 173 K were used to perform the non-linear fitting. The non-linear fitting was first performed considering the regions A and C of the experimental *E*_α_ dependency. The same fitting was then performed separately considering the regions B and C only. The values of *K*_g_ are consequently presented in Table 1 for the two materials.

According to the values of the correlation coefficient *r*^2^ and the confidence interval *I*_c_(*K*_g_), the fits are satisfactory from a statistical point of view. The different values of *K*_g_ lead to the ratio of *K*_g_(BC)/*K*_g_(AC) ~2.6 for PTFE and ~3.2 for PTFE/SiO_2_(c)F. The ratio for PTFE is correlated with the theoretical ratio of Kg_III_/Kg_II_ = 2 corresponding to a transition between regimes II and III. This transition was also highlighted in many polymeric materials, such as poly(pivalolactone), [49] high molecular iPP [50], and poly(ethylene succinate) [51]. However, it is worth noting here that the value of *K*_g_(BC)/*K*_g_(AC) for PTFE/SiO_2_(c)F is higher than the expected theoretical value of the Kg_III_/Kg_II_ ratio. Several studies mention a similar variation between the theoretical ratio and the experimental ratio that is attributed to a transition between regimes II and III [52]. Such amplitude of variation was also observed in polyethylene [40] and is justified by the involvement of multiple parameters in the crystallization process, such as nucleation rate, diffusion rate, or secondary crystallization. Additionally, the results show that the value of *K*_g_(AC) for PTFE/SiO_2_(c)F is lower than the value of *K*_g_(AC) for PTFE. This indicates that the energy barrier of nucleation is lower in the presence of nanosilica clusters for the temperatures close to melting, where the nucleation is rate-determining. This result is corroborated with the temperatures of PTFE/SiO_2_(c)F crystallization peak that appear to be higher than neat PTFE for the temperatures close to melting. The enhancement of nucleation is also correlated with the high number of nucleation centers observed in the presence of nanosilica clusters. However, the value of *K*_g_(BC) is similar for both samples. In the temperature range corresponding to region BC the nucleation rate is high (i.e., less rate-determining) and the presence of nanosilica clusters does not affect the contribution of nucleation in the crystallization process.

In order to investigate the effect of nanosilica clusters on the diffusion of crystals chains in the PTFE, the values of *K*_g_ and U* were simultaneously evaluated for both samples. The evaluation was consequently performed in the region BC, where the diffusion is rate-determining. The fit of PTFE activation energy dependency [9] and PTFE/SiO_2_(c)F activation energy dependency leads to the values of U* and *K*_g_ presented in Table 2.

In this case (Table 2), the statistical quality of the fit is higher, as attested by higher values of *r*² compared to the case where U* was arbitrary fixed to the classical value of 6270 J·mol^−1^ (Table 1). According to the results, the value of U* is clearly higher for the nanocomposite and confirms that the diffusion of crystal chains is hampered in the presence of nanosilica clusters when the crystallization is performed under very fast cooling rates.

The temperature dependences of activation energy obtained from DSC and FSC data were fitted by one single fitting curve during the evaluation of AC and BC regions. Consequently, a single set of *K*g and U* parameters were used to describe satisfactorily the fast and slow crystallization behaviors of the PTFE matrix. On this wide temperature scale, the crystallization kinetics of PTFE in the presence of the nanosilica clusters share common dynamics. A previous study highlighted similar behavior for the gelation kinetics of gelatin gels that shares common dynamics occurring on different time scales [53].

## 3. Materials and Methods

### 3.1. Materials

All of the products were provided by Aldrich Chemical Co. (Saint-Louis, MI, USA) and were used as received. PTFE (particle size 1 µm; m.p. = 321 °C; density 2.15 g·mL^−1^ at 25 °C) was employed as the polymer matrix. Tetraethoxyfluorosilane (TEFS, Aldrich number: COM448662895) was used to functionalize the nanosilica clusters surface with fluorine entities.

### 3.2. Nanosilica Clusters Synthesis

The nanosilica clusters (SiO_2_(c)) synthesis was conducted according to the nucleation-and-growth process described by Iler [54] and Parneix et al. [55]. The neutralization of an aqueous sodium silicate solution was performed with sulfuric acid, as indicated in the equation below:

(1) $$3.4SiO_{2}/Na_{2}O+H_{2}SO_{4}3.4SiO_{2}+Na_{2}SO_{4}+H_{2}O$$

First, sulfuric acid ([H_2_SO_4_] = 17 g·kg^−1^) and dilute sodium silicate solution ([SiO_2_] = 2.5 g·kg^−1^) were blended until a pH of ~9 to allow the nucleation of silica particles. The nucleation rate was controlled by temperature ranging within 60–90 °C while using a constant stirring at 250 rpm. The growth of silica particles from the nuclei was then performed by the simultaneous addition of a sodium silicate solution ([SiO_2_] = 39 g·kg^−1^) and a sulfuric acid solution ([H_2_SO_4_] = 17 g·kg^−1^). The control of addition rate leads to a pH maintained at 9 and to a temperature kept at 90 °C. After cooling to room temperature, deionized water was used to wash the mixture, allowing removal of sodium sulfate and other ions. In the end, the dispersion of nanosilica was concentrated until a weight fraction of ~0.05, displaying a final pH of ~9 and a final ionic strength of ~5·10^−3^ M.

The sodium alkoxide functions were consequently transformed into hydroxyl functions onto the silica surface by stirring the silica with sulfuric acid solution ([H_2_SO_4_] = 80 g·L^−1^) until the pH displays a value of ~0.5. In order to set the equilibrium defined by Equation (2), several washes were performed using deionized water until the pH displayed a value of ~3.

(2) $$SiO^{-}+H^{+}=SiOH$$

The value of pH located in the range 0.5–3 allows then the transformation of SiO^−^ in SiOH functions.

The morphology of nanosilica clusters (SiO_2_(c)) was investigated by TEM analysis and is displayed in Supplementary Materials. The synthesized nanoparticles are clustered with each other and a fine layer of silicate envelops each unit in order to create a silica network. According to the study of Parneix et al. [55], this silica displays a hydroxyl groups surface density of 5 OH·nm^−2^ and a specific surface of ~180 m^2^/g.

### 3.3. Surface Modification of Nanosilica Clusters

**Scheme 1:**
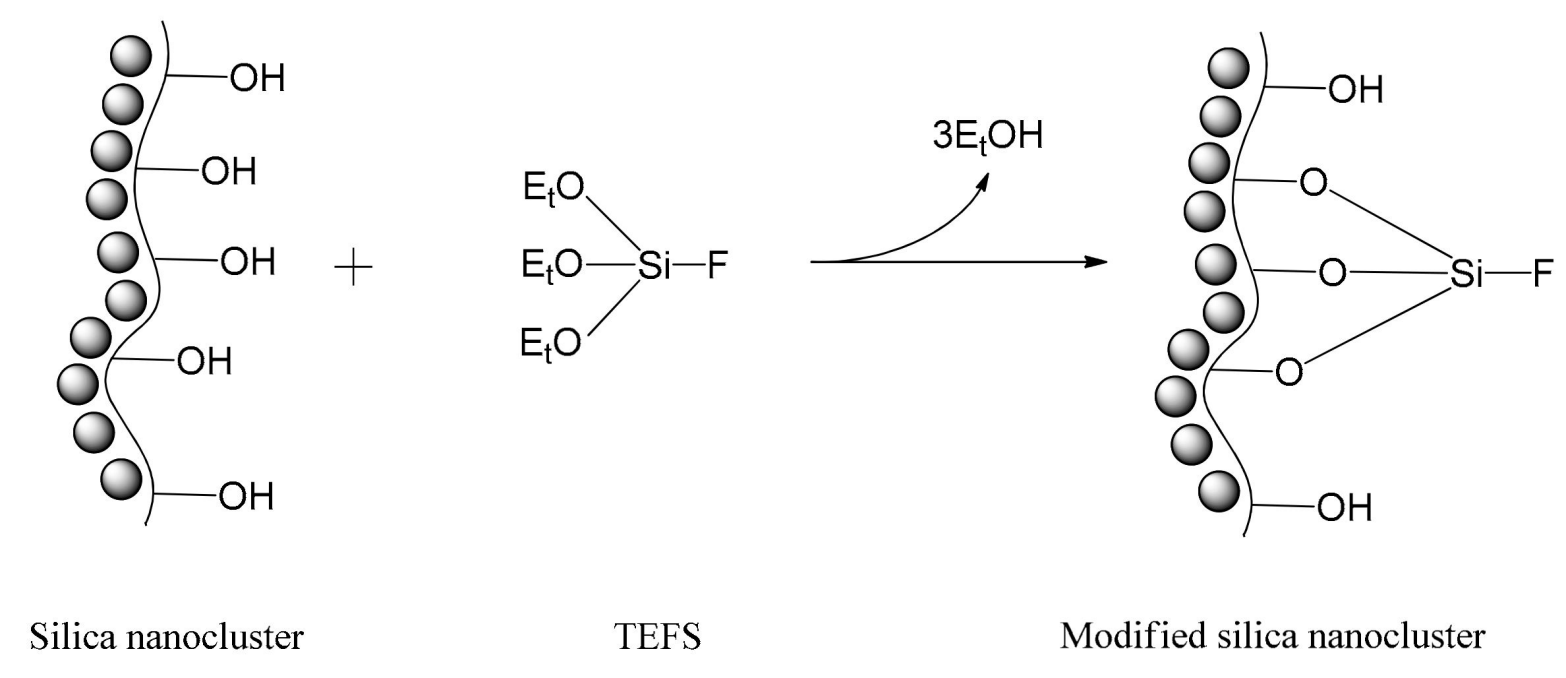
Surface modification of nanosilica clusters from SiO_2_(c) towards SiO_2_(c)F.

Several studies in the literature carried out the modification of silica surface using fluorine agents [36,56,57]. Consequently, the silica surface was modified in the present work with using TEFS, as presented on Scheme 1.

The modification was performed with a dry silica/TEFS molar ratio of ~2 in order to avoid the formation of an excessive coating layer onto the silica that may induce a decrease in specific surface and pore diameter. The blend was magnetically stirred in propanol during 1 h at ambient temperature. The fluorinated nanosilica clusters were then separated from the solution by centrifugation (3500 rounds·min^−1^) and thoroughly washed several times with propanol to eliminate the unreacted TEFS and the ethanol produced by the reaction. In order to avoid aggregation, the modified nanosilica clusters (SiO_2_(c)F) were kept into the minimum of propanol before the elaboration of the nanocomposite.

### 3.4. Elaboration of PTFE/SiO_2_(c)F Nanocomposite

The SiO_2_(c)F solvated in the propanol was placed with the PTFE in a round flask at a weight ratio of PTFE/SiO_2_(c)F = 90/10. In order to evaporate the propanol, the blend was heated at 100 °C during 2 h. The residual sample was disposed into an oven at the temperature of 300 °C during 10 min to reach the melted state. The modified nanosilica was then mechanically dispersed in the melted polymer matrix. Finally, the sample was slowly cooled until reaching ambient temperature.

### 3.5. Experimental Techniques

FTIR spectroscopy was performed on a Perkin Elmer Spectrum BX II spectrometer (Wellesley, MA, USA). Each spectrum was recorded with a total of 64 scans ranging from 4000–400 cm^−1^ and with a resolution of 4 cm^−1^. Prior to the analysis, SiO_2_(c) and SiO_2_(c)F samples were dried under vacuum at 60 °C overnight and dispersed in KBr powder. This powder was placed on a frame and the FTIR analysis was conducted in diffuse reflection mode.

All Solid-state NMR spectra were obtained on a Bruker Avance-400 (Billerica, MA, USA) MHz NMR spectrometer operating at a ^19^F and ^29^Si resonance frequency of 376.7 MHz and 79.5 MHz, respectively. About 100 mg of samples were placed in zirconium dioxide rotors of 4-mm outer diameter and spun at a Magic Angle Spinning rate of 10 kHz in a commercial Bruker Double-bearing probe. ^29^Si CPMAS experiments were performed with Cross Polarization (CP) technique [58] using a ramped 1H-pulse starting at 100% power and decreasing until 50% during the contact time (5 ms) in order to circumvent Hartmann-Hahn mismatches [59,60]. To improve the resolution, a dipolar decoupling GT8 pulse sequence [61] was applied during the acquisition time. ^19^F Single Pulse Experiment (SPE) was performed with a 90° pulse of 3.4 µs and 12.5 ms of acquisition time. To obtain a good signal-to-noise ratio, 2K scans were accumulated using a delay of 2s in ^29^Si CPMAS experiment, and 256 scans with a delay of 3s in ^19^F SPE experiment. The ^29^Si and ^19^F chemical shifts were referenced to tetramethylsilane and CFCl_3_, respectively.

The morphology of nanosilica clusters and their dispersion was investigated by Transmission Electron Microscopy (TEM). The TEM images were obtained with a JEOL JEM-1400 using an accelerator voltage of 120 kV. Samples were cut with an ultramicrotome to form ultrathin sections (~80 nm) of each material.

Scanning Electron Microscopy (SEM) was employed to observe the crystal morphologies of the neat PTFE and PTFE/SiO_2_(c)F samples. A microscope JEOL 6700F equipped with a field emission gun and an electron beam voltage set at 1 kV was employed for the observations. A silver colloidal paste was used to mount the samples on the sample holder. The samples surface was then coated with gold and palladium prior to any observation.

DSC measurements were operated via a heat flux DSC 1 from Mettler-Toledo. The calibrations of temperature, enthalpy, and tau lag were steadily done by using indium and zinc standards. Samples of ~4 mg were placed in 40 μL aluminum crucible and hermetically sealed. The experiments were conducted under an N_2_ atmosphere (80 mL·min^−1^) in order to prevent any thermo-oxidative degradation. The Hoffman-Weeks routine [31] was used to calculate the equilibrium melting temperature then estimated at the value of *T*^0^_m_ ~324 °C for both PTFE and PTFE/SiO_2_(c)F. Each DSC run was conducted in a similar way for each material. The samples were first heated at 360 °C (i.e., *T*^0^_m_ + 36 °C) during 5 min. At this temperature, the samples reach the molten state and the thermal history is erased. Then the samples were cooled from 360 to 100 °C using cooling rates ranging from 1–20 K·min^−1^ (respectively from 0.0167 to 0.3333 K·s^−1^).

FSC measurements were recorded on Flash DSC1 from Mettler-Toledo using the UFS1 chip calorimeter. The details about the instrumental setup and chip calibration can be found elsewhere [62,63,64,65]. The operating conditions employed in the present work, the consideration of thermal lag phenomena, and the measurement of sample size were conducted as indicated in our previous study [9]. The sensors were firstly conditioned and temperature-corrected as indicated by the instrument specifications. Each sample was placed directly on the sample area of the sensor, whereas the reference area remains free. Several heating and cooling scans were then performed to obtain a uniform sample that remains stuck to the sensor. This procedure also allows us to optimize the surface contact between the sample and the sensor. In order to investigate the crystallization from the melt, the samples were firstly heated at 360 °C (i.e., *T*^0^_m_ + 36 °C) and held 30 s at this temperature to erase their thermal history. The samples were then cooled from 360 to 100 °C using various cooling rates ranging from 500–5000 K·s^−1^. Crystallization temperature measured for different cooling scans available from DSC and FSC was arbitrarily chosen as the temperature corresponding to the peak maximum. The mass of the samples was about 27 ng in accordance to FSC technical specifications [63]. The sample mass was quantitatively deduced by comparing the melting enthalpy obtained in conventional DSC (J·g^−1^) with the melting enthalpy obtained in FSC (J). In both cases the samples were previously crystallized using the same cooling rate of 0.333 K·s^−1^ (i.e., 20 K·min^−1^) and consequently display comparable crystalline parts. In order to obtain the effective activation energy of crystallization, the computations of kinetics parameters were performed using the cooling rates of 0.0167, 0.0333, 0.0833 and 0.3333 K·s^−1^ by DSC and 500, 1000, 2000, 5000 K·s^−1^ by FSC.

### 3.6. Theoretical Approaches

#### 3.6.1. Kinetics

DSC is widely employed to study the crystallization kinetics of polymers, however, crystallization kinetics investigated via FSC technique are less apparent in the literature. In the present study, the macroscopic rate of crystallization can be linked to the rate of heat release measured by both DSC and FSC. The relative extent of crystallization at time *t*, *α*_t_, can be then computed according to Equation (3)

(3) $$\alpha_{t}=\frac{\int_{0}^{t}\left(dH/dt\right)\;dt}{\int_{0}^{\infty }\left(dH/dt\right)\;dt}=\frac{\alpha_{c\left(t\right)}}{\alpha_{c\left(\infty \right)}}$$

where *α*_c(t)_ and *α*_c(__∞)_ are the extent of crystallization at time *t* and at the time *t* → ∞ corresponding to the end of crystallization respectively. The general form of the basic rate equation is usually written as [66]:

(4) $$\frac{d\alpha }{dt}=k\left(T\right)\;f\left(\alpha \right)$$

where f(α) is the function representing the reaction model related to the crystallization mechanism. Arrhenius law gives the dependence of the rate coefficient with temperature:

(5) $$k\left(T\right)=A\;e^{-E/RT}$$

where *E* is the activation energy, *A* the pre-exponential factor and R the universal gas constant.

#### 3.6.2. Advanced Isoconversional Kinetic Analysis

In order to take into account the variation of *E* in the computation of the temperature integral and to overcome the drawbacks of integral methods, advanced isoconversional methods have been developed [67,68]. The advanced isoconversional methods appear to be among the most reliable kinetic methods employed for the treatment of data issued from thermoanalysis [42,66,69]. Indeed, the isoconversional methods allow us to calculate kinetic parameters without any assumption on the crystallization or reaction mechanism and are applicable to any temperature programs. These methods can be applied on crystallization kinetics data and give the dependence of the apparent activation energy *E*_α_ on the relative degree of crystallinity *α*. Via this *E*_α_ dependency, the treatment and detection of multi-step kinetics is possible allowing meaningful mechanistic and kinetic analyses. The *E*_α_ value is associated to the value that minimizes the function [70]:

(6) $$\Phi \left(E_{\alpha }\right)=\overset{n}{\underset{i=1}{\sum }}\overset{n}{\underset{j\neq i}{\sum }}\frac{J\left[E_{\alpha },T_{i}\left(t_{\alpha }\right)\right]}{J\left[E_{\alpha },T_{j}\left(t_{\alpha }\right)\right]}$$

where *J* is evaluated over small intervals of *E* variation:

(7) $$J\left[E_{\alpha },T_{i}\left(t_{\alpha }\right)\right]\equiv \int_{t_{\alpha -\Delta \alpha }}^{t_{\alpha }}exp\left[\frac{-E_{\alpha }}{RT_{i}\left(t\right)}\right]dt$$

According to the method proposed by Sbirrazzuoli, the values of *E*_α_ were computed using an internally generated software for each value of α lying in between 0.02–0.98, using a step of 0.02 [71,72,73,74]. The application of the Lagrangian algorithm on the computations led to an accurate interpolation of the integrated α-T curves and allowed to increase the number of points of FSC data recorded for fast crystallization of PTFE. Isoconversional kinetic analysis appears consequently to be a powerful concept to obtain important information on the related mechanisms [42] and was applied in the present work to the non-isothermal crystallization data obtained from both regular DSC and FSC measurements. In the present study, this computation is referred to as a non-linear method (NLN).

#### 3.6.3. Hoffman-Lauritzen Theory of Crystallization

According to the method proposed by Vyazovkin and Sbirrazzuoli [47], the parameters U* and *K*_g_ of the Hoffman–Lauritzen theory [32], corresponding to the activation energy of the segmental jump (related to the diffusion process) and to the activation energy of the nucleation of a crystal with a specific size respectively, can be evaluated by the fit of the resulting *E*_α_ vs. *T*-dependence to the following equation:

(8) $$E_{\alpha }\left(T\right)=U^{*}\frac{T^{2}}{{\left(T-T_{\infty }\right)}^{2}}+K_{g}R\frac{{\left(T_{m}^{0}\right)}^{2}-T^{2}-T_{m}^{0}T}{{\left(T_{m}^{0}-T\right)}^{2}T}$$

With *T*_∞_ the hypothetical temperature where motion associated with viscous flow ceases (i.e., the temperature taken 30K below the glass transition temperature, *T*_g_), R the universal gas constant and *T*^0^_m_ the equilibrium melting temperature.

The kinetic parameter *K*_g_ presented in Equation (8) can be defined as below:

(9) $$K_{g}=\frac{nb\sigma \sigma_{e}T_{m}}{\Delta h_{f}k_{B}}$$

where *b* is the surface nucleus thickness, *σ* is the free energy of lateral surface, *σ*_e_ is the free energy of fold surface, Δ*h*_f_ is the heat of fusion per unit volume of crystal, *k*_B_ is the Boltzmann constant, and *n* takes the value 4 for crystallization regime I and III, and 2 for regime II. The dependencies of *E*_α_ vs. *T* obtained in the present study were fitted to Equation (8). Origin 8.5 software was used to perform the non-linear fitting to the experimental *E*_α_-dependence.

## 4. Conclusions

The present study investigates the influence of fluorinated nanosilica clusters on the crystallization of PTFE under various cooling rates, and originally highlights the variation of the effect induced by the filler (i.e., promotion of nucleation and hindrance of diffusion) according to the cooling rates considered. The combination between DSC and FSC measurements allowed us to observe the crystallization from the melt of neat PTFE and PTFE/SiO_2_(c)F in a wide range of temperatures, imparting to this work an original investigation that stands out from conventional approaches. The silica promotes the crystallization at slow cooling rates by inducing a nucleating effect but appears to hinder the crystallization at fast cooling rates. The contribution of this filler as a nucleating agent is confirmed by SEM observations showing that the presence of nanosilica clusters leads to a higher number of nucleation centers, but also allows the formation of stable and more perfect crystals even when high cooling rates are employed, and promotes the formation of two-dimensional structures. The advanced isoconversional kinetic analysis was applied to the crystallization of neat PTFE and PTFE/SiO_2_(c)F occurring on a large temperature range, leading to an *E*_α_ dependency depicting negative increasing values associated with the anti-arrhenian behavior of crystallization from the melt. The application of Hoffman-Lauritzen theory on *E*_α_ dependency allowed us to estimate the nucleation parameter *K*_g_ from the *E*_α_ dependency of neat PTFE and PTFE/SiO_2_(c)F crystallization. The estimation of *K*_g_ values of regions AC and BC indicated a transition from regime II to regime III for both materials. The obtained value of *K*_g_(AC) is lower for PTFE/SiO_2_(c)F and then confirms the nucleation effect of the silica for the temperatures close to the melting. However, this nucleation effect was not apparent for the temperatures located further from the melting, where the presence of nanosilica clusters hinders the diffusion of crystal chains and consequently hampers the crystallization.

## Figures and Tables

**Table 1:** Estimation of *K*_g_ parameter for PTFE and PTFE/SiO_2_(c)F.

Region	U*/J·mol^−1^	PTFE			PTFE/SiO_2_(c)F		
		10^4^ *K*_g_/K^2^	*I*_c_ (*K*_g_)/%	*r* ^2^	10^4^ *K*_g_/K^2^	*I*_c_ (*K*_g_)/%	*r* ^2^
AC	6270	3.4	~1	0.983	2.7	1.7	0.981
BC	6270	9.0	~1	0.994	8.6	2.6	0.983

**Table 2:** Estimation of U* and *K*_g_ parameters for PTFE and PTFE/SiO_2_(c)F on the BC region.

	U*/J·mol^−1^	*I*_c_ (U*)/%	10^4^ *K*_g_/K^2^	*I*_c_ (*K*_g_)/%	*r* ^2^
PTFE	5870	~37	8.7	~0.8	0.995
PTFE/SiO_2_(c)F	90334	~3.9	11.3	~1	0.997

## Supplementary Materials

The following are available online at https://www.mdpi.com/1420-3049/24/9/1797/s1, Figure S1: TEM pictures of SiO_2_(c) nanoparticles, Figure S2: Relative degree of crystallinity vs temperature during the nonisothermal crystallization from the melt of neat PTFE and PTFE/ SiO_2_(c)F obtained by DSC and FSC (b).
