# Supplementary material for: Crystallization of Polytetrafluoroethylene in a Wide Range of Cooling Rates: Nucleation and Diffusion in the Presence of Nanosilica Clusters
Source: Molecules. 2019 May 9;24(9):1797. doi: 10.3390/molecules24091797 (PMC6539400; doi:10.3390/molecules24091797)
Supplement: Supplementary file 1 [file molecules-24-01797-s001.pdf]

molecules

MDPI

SUPPORTING INFORMATION

Crystallization of Polytetrafluoroethylene in a Wide Range of Cooling Rates: Nucleation and Diffusion in the Presence of Nanosilica Clusters

Nicolas Bosq [1], Nathanaël Guigo [2,*], Jacques Persello [3] and Nicolas Sbirrazzuoli [4,*]

[1] Université Côte d'Azur, Institut de Chimie de Nice, UMR CNRS 7272, 06100 Nice, France; nicolas.bosq@univ-cotedazur.fr
[2] Université Côte d'Azur, Institut de Chimie de Nice, UMR CNRS 7272, 06100 Nice, France;
[3] Université Côte d'Azur, Institut de Physique de Nice, UMR CNRS 7010, 06100 Nice, France; jacques.persello@univ-cotedazur.fr
[4] Université Côte d'Azur, Institut de Chimie de Nice, UMR CNRS 7272, 06100 Nice, France; nicolas.sbirrazzuoli@unice.fr
* Correspondence: nathanael.guigo@univ-cotedazur.fr (N.G.); nicolas.sbirrazzuoli@unice.fr (N.S.); Tel.: +33-04-9207-6179 (N.S.)

1. Transmission Electron Microscopy……………………….………....…………......…..S1

2. Relative degree of crystallinity versus temperature..…………………………………...S2

1. Transmission Electron Microscopy

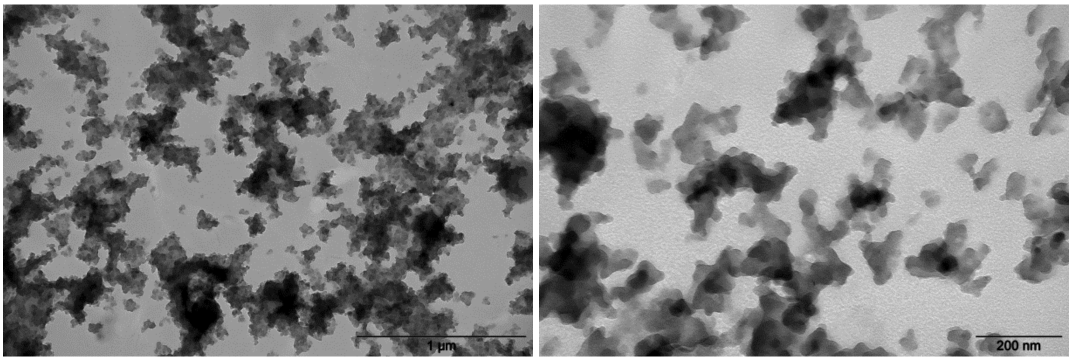

Figure 1. TEM pictures of SiO_2(c) nanoparticles.

2. Relative Degree of Crystallinity Versus Temperature

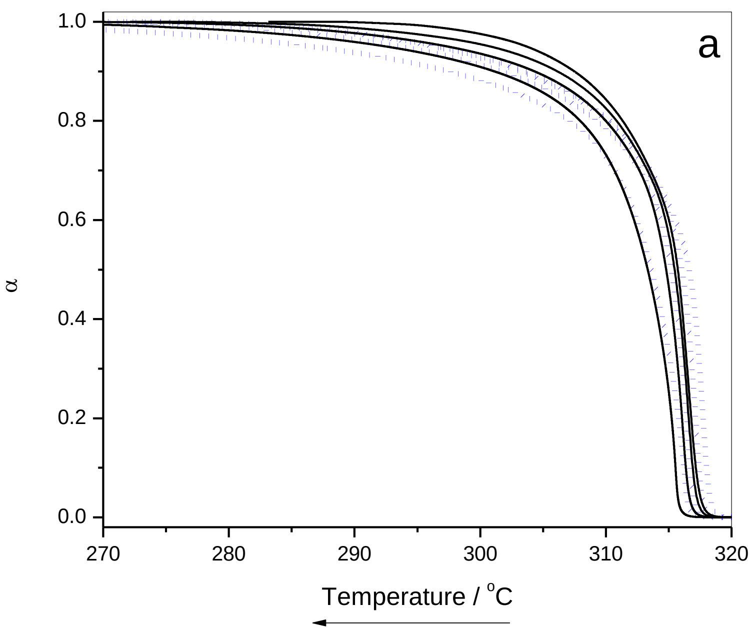

(a)

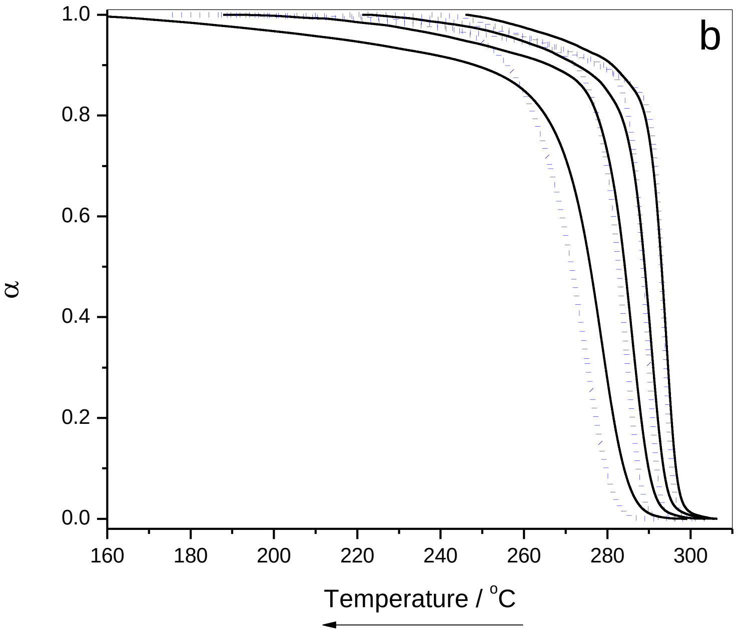

(b)

Figure 2. Relative degree of crystallinity vs temperature during the nonisothermal crystallization from the melt of neat PTFE (black, line) and PTFE/ SiO_2(c)F (blue, dot) obtained by DSC (**a**) and FSC (**b**). The cooling rate of each experiment (in $K{\cdot}s^{-1}$) is indicated by each curve. Inset: magnification of the α vs T curve at the beginning of crystallization process.
